# Unlocking the Potential: Novel NSAIDs Hybrids Unleash Chemopreventive Power toward Liver Cancer Cells through Nrf2, NF-κB, and MAPK Signaling Pathways

**DOI:** 10.3390/molecules28155759

**Published:** 2023-07-30

**Authors:** Maria Narożna, Violetta Krajka-Kuźniak, Barbara Bednarczyk-Cwynar, Wanda Baer-Dubowska

**Affiliations:** 1 Program in Cell Cycle and Cancer Biology, Oklahoma Medical Research Foundation, 825, NE 13th Street, Oklahoma City, OK 73104, USA; maria-narozna@omrf.org; 2 Department of Pharmaceutical Biochemistry, Poznan University of Medical Sciences, 4, Święcicki Street, 60-781 Poznań, Poland; vkrajka@ump.edu.pl; 3 Department of Organic Chemistry, Poznan University of Medical Sciences, 6, Grunwaldzka Street, 60-780 Poznań, Poland; bcwynar@ump.edu.pl

**Keywords:** Nrf2, NF-κB, MAPK, HCC, Ibuprofen, Ketoprofen, oleanolic acid oximes, liver cancer, NSAIDs

## Abstract

HCC is a highly aggressive malignancy with limited treatment options. In this study, novel conjugates of non-steroidal anti-inflammatory drugs (NSAIDs)—Ibuprofen and Ketoprofen—with oleanolic acid oximes derivatives (OAO) were synthesized, and their activity as modulators of signaling pathways involved in HCC pathogenesis was evaluated in normal THLE-2 liver cells, and HCC-derived HepG2 cells. The results demonstrated that conjugation with OAO derivatives reduces the cytotoxicity of parent compounds in both cell lines. In THLE-2 cells, treatment with conjugates resulted in increased activation of the Nrf2-ARE pathway. An opposite effect was observed in HepG2 cells. In the later reduction of NF-κB, it was observed along with modulation of MAPK signaling pathways (AKT, ERK, p38, p70S6K, and JNK). Moreover, STAT3, STAT5, and CREB transcription factors on protein levels were significantly reduced as a result of treatment with IBU- and KET-OAO derivatives conjugates. The most active were conjugates with OAO-morpholide. Overall, the findings of this study demonstrate that IBU-OAO and KET-OAO derivative conjugates modulate the key signaling pathways involved in hepatic cancer development. Their effect on specific signaling pathways varied depending on the structure of the conjugate. Since the conjugation of IBU and KET with OAO derivatives reduced their cytotoxicity, the conjugates may be considered good candidates for the prevention of liver cancer.

## 1. Introduction

Hepatocellular carcinoma (HCC) remains a global health burden characterized by high mortality rates and limited treatment options. Chronic liver inflammation, often driven by viral hepatitis, alcohol abuse, or nonalcoholic steatohepatitis, significantly contributes to the development and progression of HCC [1].

The nuclear factor erythroid 2-related factor 2 binding to antioxidant response element in DNA (Nrf2-ARE) and nuclear factor-kappa B (NF-κB) signaling pathways play a key role in HCC development. The Nrf2-ARE signaling pathway is responsible for the transcription of genes encoding cytoprotective proteins. Therefore, its activation at the initiation step may protect against cancer development, while NF-κB activated in response to inflammatory stimuli contribute to the promotion of the carcinogenesis process [2,3]. One of the target genes of the latter is cyclooxygenase-2 (COX-2), which overexpression characterizes several cancers, including HCC [4,5,6]. Usually, reduced activation and expression of NF-κB results in the opposite effect in the Nrf2 signaling pathway [7]. However, it has also been demonstrated that Nrf2 is often overexpressed in cancer cells, including HCC, and may lead to an enhanced invasiveness potential and chemo-and radio-resistance [8]. Therefore, inhibition of Nrf2 is highly desired at the later stages of cancer development. In addition to NF-κB and Nrf2, mitogen-activated protein kinases (MAPKs) signaling pathways, such as Janus kinase/signal transducer and activator of transcription (Jak/STAT), cyclic-AMP response element-binding protein (CREB), phosphatidylinositol-3-kinase/serine-threonine kinase AKT (PI3K/Akt), are involved in liver cancer development and inflammation responses. Dysregulation of MAPKs pathways has been implicated in liver cancer progression. Furthermore, these pathways exhibit intricate crosstalk, suggesting that targeting multiple signaling pathways holds promise as a therapeutic approach to HCC [9,10]. Due to the inflammatory background of several cancers, including HCC, non-steroidal anti-inflammatory drugs (NSAIDs) have been proposed for their prophylaxis and/or therapy [11,12,13,14].

However, although efficient, those drugs exhibit unfavorable side effects, in particular, related to the insurgence of stomach or intestinal bleeding and circulatory or cardiac deregulation [15]. Thus, natural alternatives are searched. One of them is oleanolic acid. It possesses strong hepatoprotective and anti-inflammatory potential but very low bioavailability [16,17,18,19]. Hence, a reasonable strategy is to modify its chemical structure to obtain derivatives with increased solubility, permeability, and enhanced therapeutic potential. Distinguished strategies for modifying its structure include synthesizing oximes, esters, and amides [20]. Our earlier study showed that oleanolic acid oximes (OAO) substituted at the C-17 with methyl, benzyl ester, or morpholide group significantly increases their ability to modulate the NF-κB signaling pathway [21]. Moreover, conjugation of these derivatives with NSAIDs such as aspirin, indomethacin, or diclofenac enhanced their modulating effect on key cellular signaling pathways, ultimately leading to induction of apoptosis and reduced proliferation of HCC cells and reducing tumor burden in vivo [22,23,24].

Ibuprofen (IBU) and Ketoprofen (KET) are other representatives of NSAIDs that have been used for many years due to their anti-inflammatory, analgesic, and antipyretic properties. Like many other NSAIDs, they inhibit the cyclooxygenase enzyme (COX) and act by decreasing the production of prostaglandin inflammatory precursors [25,26]. While IBU targets both COX-1 and COX-2 isozymes, KET is more specific toward COX-1 [27,28]. Their anticancer activity was proven in some types of cancer, e.g., lung cancer [29,30]. In one prospective data analysis performed in 2012, in contrast to aspirin which use was associated with reduced risk of developing HCC and death due to chronic liver diseases (CLD), IBU and KET were associated only with reduced risk of death due to CLD [31]. COX-2 gene expression is regulated by transcription factor NF-κB, the end product of NF-κB signaling pathway activation. Thus, besides the direct interaction with the enzyme, it is possible that IBU and KET might affect COX-2 gene expression. Naturally occurring oleanolic acid (OA) is effective as a hepatoprotective agent, acting mainly through reprogramming the liver to activate the Nrf2 pathway [32]. Its oximes (OAO) substituted with different groups, particularly morpholide, as showed in our earlier studies, were even more potent Nrf2 activators [22,23,24]. Moreover, they decreased the activation of the NF-κB pathway. Therefore, the conjugation of IBU and KET with OAO derivatives may increase their efficacy. Moreover, their potential combined action on the elements of the MAPKs signaling pathway may lead to the anakoinosis effect, which, by targeting the simultaneously different, but cross-talking signaling pathways, is considered an innovative anticancer approach [33].

Thus, the aim of this study was the synthesis and evaluation of the potential anticancer activity of novel OAO derivatives conjugated with IBU (IBU-OAO) and KET (KET-OAO) on HCC-derived HepG2 cells and THLE-2 normal, immortalized hepatocytes. Their effect on cell viability and ability to modulate the Nrf2-ARE, NF-κB, and MAPKs signaling pathways was evaluated in order to compare with the earlier studied conjugates and select the optimal structures of the compounds for future in vivo studies.

## 2. Results

### 2.1. Chemistry

**Figure 1:**
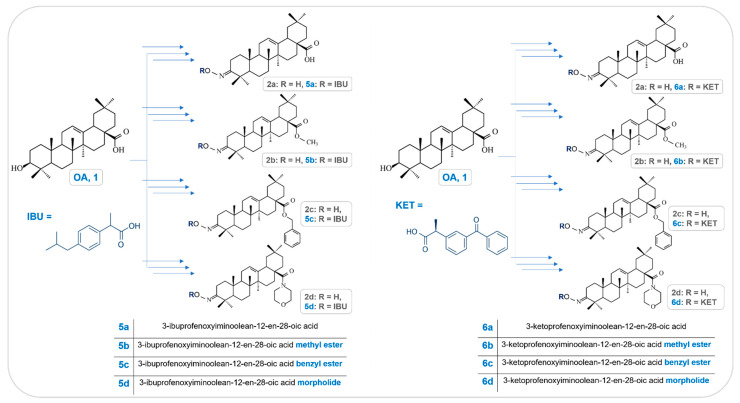
The brief scheme of synthesis and chemical structures of oleanolic acid oximes (**2a**–**d**) and the investigated conjugates with Ibuprofen (IBU-OAO) (**5a**–**d**) and Ketoprofen (KET-OAO) (**6a**–**d**).

Oleanolic acid oxime conjugates with IBU (**5a**–**d**) and KET (**6a**–**d**) were obtained using the earlier published methods [21,23,24]. Figure 1 presents a synthesis overview of the investigated IBU-OAO and KET-OAO conjugates.

### 2.2. Spectral Characteristics of the OAO Conjugates with Ibuprofen and Ketoprofen

#### 2.2.1. Conjugates of OAO and Ibuprofen (**5a**–**d**)

In the IR spectra of conjugates **5a**–**d**, the presence of an aromatic ring within the Ibuprofen moiety ([CH_3_]_2_CH-CH_2_-C_6_H_4_-CH[CH_3_]-COON=C_-3_) was confirmed by the presence of an absorption band located at ν 3335–3340 cm^−1^. The signal observed at ν 1725–1735 cm^−1^ was assigned to the C=O group within the –COO function of the Ibuprofen moiety ([CH_3_]_2_CH-CH_2_-C_6_H_4_-CH[CH_3_]-COON=C_-3_). The next characteristic absorption band, observed at ν 1720 or 1725 cm^−1^, was assigned to –N=C_-3_ of the acyloxyimino function.

The NMR analysis of conjugates **5a**–**d** confirmed the structures of these compounds.

In the ^13^C NMR spectra of the above conjugates, the signal of the C=O group within the –COO function of the Ibuprofen moiety ([CH_3_]_2_CH-CH_2_-C_6_H_4_-CH[CH_3_]-COON=C_-3_) was observed at the δ 176 ppm. Signals derived from the aromatic ring of Ibuprofen moiety were present at δ about 140, 138 (2 × C_q_), 137, 129, 128, and 127 (4 × CH) ppm. Other signals characteristic for the Ibuprofen system were observed at δ about 45 ppm (CH_2_, [CH_3_]_2_CH-CH_2_-C_6_H_4_-CH-[CH_3_]-COON=C_-3_), 41 ppm (CH, [CH_3_]_2_CH-CH_2_-C_6_H_4_-CH-[CH_3_]-COON=C_-3_), 27 ppm (CH, [CH_3_]_2_CH-CH_2_-C_6_H_4_-CH-[CH_3_]-COON=C_-3_), 22 ppm (CH_3_ × 2, [CH_3_]_2_CH-CH_2_-C_6_H_4_-CH-[CH_3_]-COON=C_-3_) and 15 ppm (CH_3_, [CH_3_]_2_CH-CH_2_-C_6_H_4_-CH-[CH_3_]-COON=C_-3_). Four signals characteristic of the oleanane system were noticed at δ about 172 ppm (C_q_, C-3), 144 ppm (C_q_, C-13), 122 ppm (CH, C-12), and 47 ppm (C_q_, C-17).

In the ^1^H NMR spectra of oleanolic acid oximes conjugates with Ibuprofen, the protons of an aromatic system within Ibuprofen moiety formed two signals: a doublet of doublets (present at δ about 7.2 ppm) and a doublet (present at δ about 7.1 ppm). The other six signals characteristic of the Ibuprofen system were observed at δ about 3.8 ppm (a quartet for [CH_3_]_2_CH-CH_2_-C_6_H_4_-CH-[CH_3_]-COON=C_-3_), 2.5 ppm (a doublet for [CH_3_]_2_CH-CH_2_-C_6_H_4_-CH-[CH_3_]-COON=C_-3_), 2.0 ppm (a triplet of triplets for [CH_3_]_2_CH-CH_2_-C_6_H_4_-CH-[CH_3_]-COON=C_-3_), 1.5 ppm (a doublet for [CH_3_]_2_CH-CH_2_-C_6_H_4_-CH-[CH_3_]-COON=C_-3_), 1.2, and 1.1 ppm (two doublets for ([CH_3_]_2_CH-CH_2_-C_6_H_4_-CH-[CH_3_]-COON=C_-3_). Two signals characteristic of the oleanane system were present at δ about 5.3 ppm (a triplet for C_12_-H) and 2.9 ppm (a doublet of doublets for C_18_-H_β_).

#### 2.2.2. Conjugates of OAO and Ketoprofen (**6a**–**d**)

In the IR spectra of conjugates **6a**–**d**, the presence of aromatic rings within the Ketoprofen moiety (C_6_H_5_-CO-C_6_H_4_-CH[CH_3_]-COON=C-_3_) was confirmed by the presence of an absorption band located at ν 3330–3335 cm^−1^. The signal observed at ν 1730–17,405 cm^−1^ was assigned to the C=O group within the –COO function of the Ketoprofen moiety (C_6_H_5_-CO-C_6_H_4_-CH[CH_3_]-COON=C-_3_). The next characteristic absorption band, observed at ν 1720 or 1725 cm^−1^, was assigned to –N=C_-3_ of the acyloxyimino function.

The NMR analysis of conjugates **6a**–**d** confirmed the structures of these compounds.

In the ^13^C NMR spectra of the above conjugates, the signal of the C=O group that joined two aromatic rings within the Ketoprofen moiety (C_6_H_5_-CO-C_6_H_4_-CH[CH_3_]-COON=C_-3_) was observed at δ about 197 ppm. The signal of the C=O group within the –COO function of the Ketoprofen moiety (C_6_H_5_-CO-C_6_H_4_-CH[CH_3_]-COON=C_-3_) was observed at the δ 176 ppm. Signals derived from an aromatic ring of Ketoprofen moiety were present at δ about 141 ppm (C_q_), 138 ppm (C_q_), 131 ppm (CH), 129 ppm (CH), 129 ppm (CH), 129 ppm (CH, C_6_H_5_-CO-C_6_H_4_-CH[CH_3_]-COON=C_-3_), 137 ppm (C_q_), 132 ppm (CH), 130 ppm (2 × CH) and 128 ppm (2 × CH, C_6_H_5_-CO-C_6_H_4_-CH[CH_3_]-COON=C_-3_). The other two signals characteristic of the Ibuprofen system were observed at δ about 44 ppm (CH, C_6_H_5_-CO-C_6_H_4_-CH[CH_3_]-COON=C_-3_), and 18 ppm (CH_3_, C_6_H_5_-CO-C_6_H_4_-CH[CH_3_]-COON=C_-3_). Four signals characteristic of oleanane system were noticed at δ about 172 ppm (C_q_, C-3), 144 ppm (C_q_, C-13), 122 ppm (CH, C-12), and 47 ppm (C_q_, C-17).

In the ^1^H NMR spectra of conjugates **6a**–**d** with Ketoprofen, the protons of aromatic systems within Ketoprofen moiety (C_6_H_5_-CO-C_6_H_4_-CH-[CH_3_]-COON=C_3_) formed four signals: a doublet of doublets located at δ about 7.8 ppm, a doublet observed at δ about 7.7 ppm, a triplet observed at δ about 7.6 ppm and a multiplet at δ about 7.5–7.4 ppm. Two more signals derived from Ketoprofen moiety were present at δ about 4 ppm 3.98 (a quartet for C_6_H_5_-CO-C_6_H_4_-CH-[CH_3_]-COON=C_3_) and 1.6 ppm (a doublet for C_6_H_5_-CO-C_6_H_4_-CH-[CH_3_]-COON=C_3_). Two next signals, characteristic of the oleanane system, were located at δ about 5.30 (a triplet for C_12_-H) and δ about 3 ppm (a doublet of doublets for C_18_-H_β_).

### 2.3. Conjugation with OAO Derivatives Decreases the Cytotoxicity of Ibuprofen and Ketoprofen

**Figure 2:**
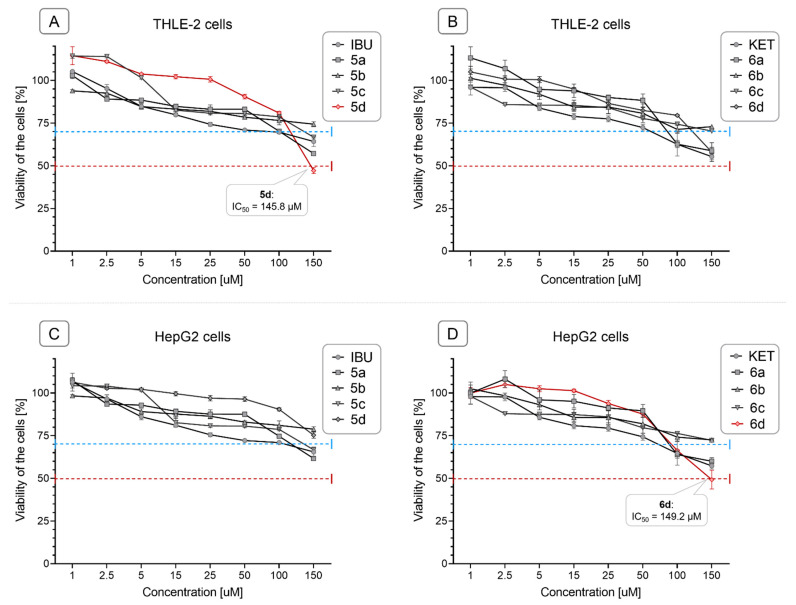
The effect of the IBU, KET, IBU–OAO conjugates (**5a**–**d**) and KET–OAO conjugates (**6a**–**d**) on the viability of THLE-2 (**A**,**B**) and HepG2 (**C**,**D**) cells (after 24 h incubation). The red dashed line on the graphs represents the IC_50_ level (half maximal inhibitory concentration), the concentration at which the compound is able to reduce the viability of cells by half. The blue dashed line in the graphs represents the concentration of compounds that ensures approximately 70% cell viability. Data (mean ± SEM) from three separate experiments are presented.

The viability of the immortalized hepatocytes line THLE-2 and HCC-derived HepG2 cells was assessed using the MTT assay after treatment with IBU, KET, and their conjugates at a concentration range of 1–150 µM. As depicted in Figure 2A–D, the conjugation of IBU and KET with OAO resulted in a reduction of cytotoxicity in both cell lines. Notably, the majority of the conjugates exhibited lower cytotoxicity compared to the parent compounds (IBU and KET). However, IBU-OAO morpholide conjugate **5d** in THLE-2 cells and OAO-KET morpholide conjugate **6d** in HepG2 cells displayed a slight deviation from this trend, as they reached the IC_50_ values, albeit with a marginal difference, 145.8 µM, and 149.2 µM, respectively.

Based on the results obtained from the MTT assay, subsequent assays were performed using a concentration of 30 µM, which ensured more than 70% cell viability. This concentration was chosen to further investigate the effects of the compounds on subsequent molecular and cellular processes.

### 2.4. Conjugation of Ibuprofen and Ketoprofen with OAO Derivatives Increases Activation and Expression of Nrf2 in THLE-2 Cells but Reduces in HepG2 Cells

**Figure 3:**
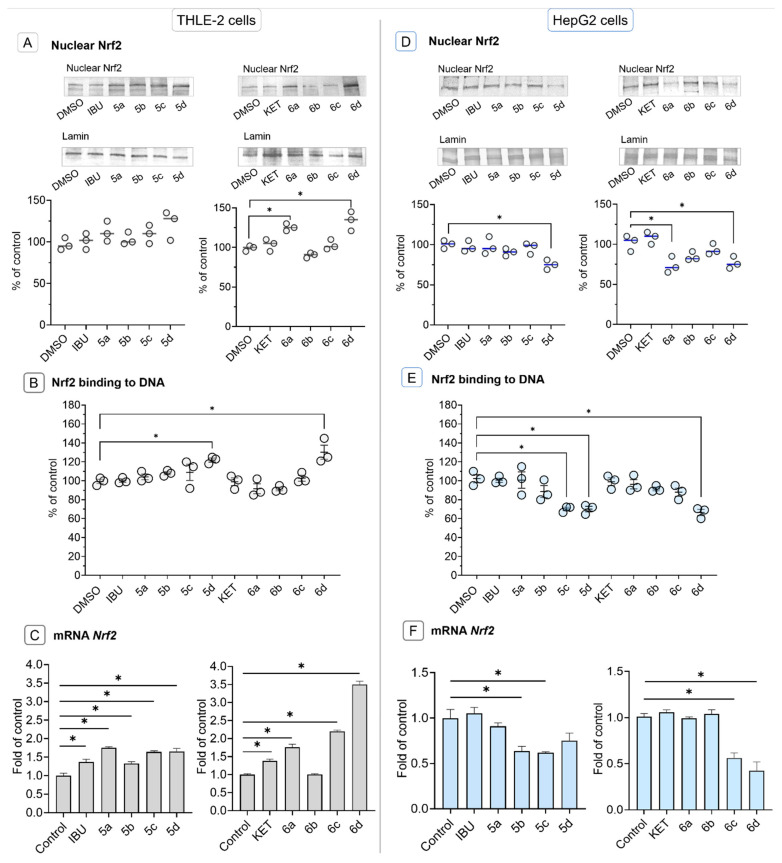
The effect of the IBU, KET, IBU–OAO conjugates (**5a**–**d**) and KET–OAO conjugates (**6a**–**d**) at concentrations of 30 µM on Nrf2 activation and expression in THLE-2 cells (**A**–**C**) and HepG2 cells (**D**–**F**) after 24 h treatment. Panels (**A**,**D**) present nuclear protein levels of Nrf2, panels (**B**,**E**) Nrf2 binding to DNA, and panels (**C**,**F**) mRNA transcript panels. Representative Western immunoblots are presented above the graphs. The sequence of the bands corresponds to the sequence of the bars in the graph. Lamin, a major component of the nuclear lamina, was used for normalizing nuclear protein levels in Western blot analysis. The values (means ± SEM) were calculated as protein levels compared to control cells (expression equal to 100%). Activated Nrf2 (**B**,**E**) was assessed in terms of the amount of Nrf2 contained in the DNA-binding complexes extracted from the nuclei isolated from the cells and calculated compared to control cells set to 100%. The values of mRNA level (**C**,**F**) were calculated as the relative change in comparison to control cells (expression equal to 1). * Significantly different from control: DMSO-treated cells, *p* < 0.05. Student’s *t*-test was used to assess the statistical significance.

Activation of Nrf2 either by avoiding degradation by Keap1 protein or phosphorylation requires translocation from the cytosol to the nucleus [2]. In THLE-2 cells, treatment with KET-OAO conjugates **6a** and **6d** resulted in increased levels of nuclear Nrf2 protein (Figure 3A). Furthermore, treatment with IBU-OAO morpholide **5d** and KET-OAO morpholide **6d** led to an enhanced binding to the antioxidant response element (ARE) sequence, as measured by the amount of Nrf2 contained in the DNA binding complex to the ARE sequence. The increase in binding was observed to be 25% and 30%, respectively. The oligonucleotides containing ARE consensus-binding site (5′-GTCACAGTGACTCAGCAGAATCTG-3′) for Nrf2 were immobilized on microplates as bait. The binding of the active form of Nrf2 to its target sequence leads to the transcription of genes encoding cytoprotective proteins neutralizing, e.g., reactive oxygen or electrophilic species [34].

To investigate the effect on Nrf2 gene expression at the transcript level, the levels of Nrf2 mRNA were assessed. Treatment with all IBU-OAO conjugates resulted in increased expression of the Nrf2 gene. In normal hepatocytes, IBU-OAO conjugates **5a** and **5d** demonstrated the most pronounced effect showing a 1.75-fold and 1.65-fold increase in Nrf2 gene expression, respectively. Among the KET-OAO hybrids, the most effective was compound **6d,** i.e., the morpholide derivative conjugated with KET, showing a 3.5-fold increase compared to the control (Figure 3C).

In contrast, in HepG2 cells, treatment with IBU-OAO **5d**, KET-OAO **6a**, and **6d** led to a reduction in the level of nuclear Nrf2 protein (Figure 3D). Similarly, the binding of Nrf2 to the ARE sequence was reduced by IBU-OAO **5c**, **5d**, and KET-OAO **6d** (Figure 3E). Consistent with the protein level results, the Nrf2 mRNA level was diminished after treatment with IBU-OAO **5b**, **5c**, **5d**, and **6a**, ranging from 25% to 57% reduction (Figure 3F). Notably, the most effective modulator of Nrf2 activation and expression in both cell lines was the KET-OAO conjugate **6d**.

These findings demonstrate that the conjugation of IBU and KET with OAO increases the activation and expression of Nrf2 in THLE-2 cells while producing opposing effects in HepG2 cells. The KET-OAO conjugate **6d** exhibited the most significant modulation of Nrf2 activation and expression.

### 2.5. Ibuprofen and Ketoprofen—OAO Conjugates Reduce the Activation and Expression of NF-κB and COX-2 in HepG2 Cells

**Figure 4:**
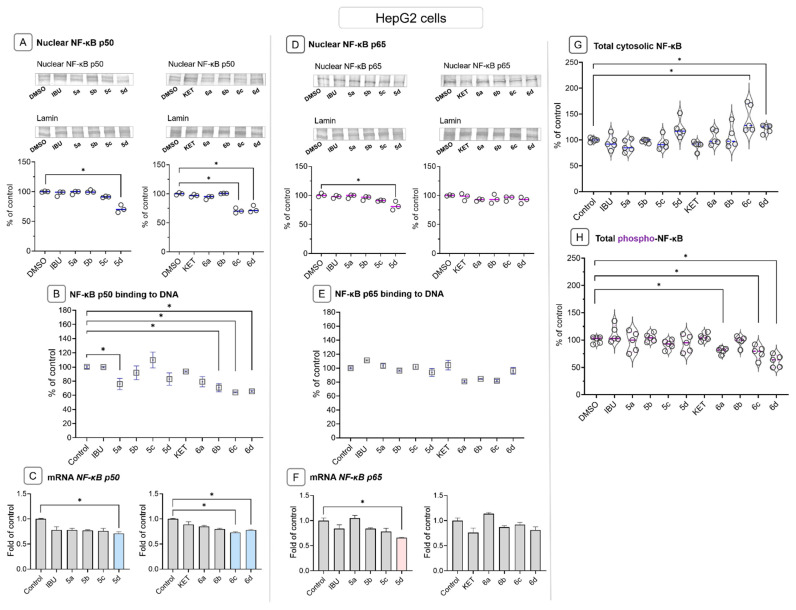
The effect of the IBU, KET, IBU–OAO conjugates (**5a**–**d**) and KET–OAO conjugates (**6a**–**d**) at concentrations of 30 µM on NF-κB activation and expression in HepG2 cells after 24 h treatment. Panels (**A**,**D**) show NF-κB p50 and p65 subunits nuclear protein levels, binding to DNA (**B**,**E**) and mRNA transcripts panels (**C**,**F**). Panels (**G**,**H**) show the total cytosolic NF-κB and the total phosphorylated NF-κB protein levels. Representative Western immunoblots are presented above the graphs. The sequence of the bands corresponds to the sequence of the bars in the graph. Lamin was used for normalizing nuclear protein levels in Western blot analysis. The values (means ± SEM) were calculated as protein levels compared to control cells (expression equal to 100%). Activated NF-κB p50 and p65 (**B**,**E**) were assessed in terms of the amount of NF-κB subunits contained in the DNA-binding complexes extracted from the nuclei isolated from the cells and calculated compared to control cells set to 100%. The values of mRNA level (**C**,**F**) were calculated as the relative change in comparison to control cells (expression equal to 1). The cytosolic NF-κB and phospho-NF-κB (**G**,**H**) were assessed using the MAGPIX^®^ system, and the results from five independent measurements are presented as a fold of fluorescence intensity obtained in control cells. * Significantly different from control: DMSO-treated cells, *p* < 0.05. Student’s *t*-test was used to assess the statistical significance.

To assess the effect of IBU-OAO and KET-OAO conjugates on NF-κB activity, the binding of p50 and p65 subunits to their consensus site and their nuclear levels was examined. Treatment with IBU-OAO conjugate **5a** resulted in a reduction in p50 binding (~24%), while treatment with KET conjugates **6b**, **6c**, and **6d** decreased p50 binding by approximately 30–35%. The binding of the p65 subunit was not significantly affected (Figure 4B,E). Moreover, compounds **5d**, **6c,** and **6d** led to a reduction in the nuclear level of the p50 subunit protein (Figure 4A), increasing the content of the NF-κB whole protein complex in the cytosol (Figure 4G). Furthermore, treatment with KET-OAO conjugates, specifically compounds **6a**, **6c**, and **6d**, decreased the level of phosphorylated NF-κB (Figure 4H).

At the transcript level, compounds **5d** and **6d** reduced the expression of the NF-κB p50 subunit gene, while compound **5d** decreased the expression of the NF-κB p65 subunit gene by approximately 36% (Figure 4C,F).

**Figure 5:**
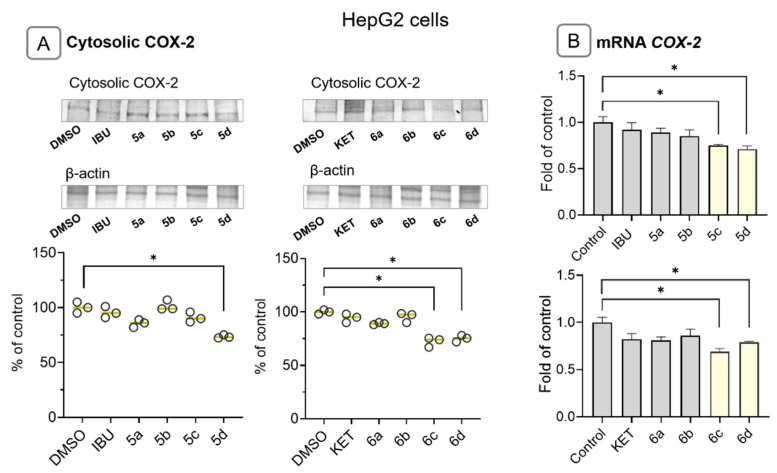
The effect of the IBU, KET, IBU–OAO conjugates (**5a**–**d**) and KET–OAO conjugates (**6a**–**d**) at concentrations of 30 µM on the COX-2 protein (**A**) and COX-2 mRNA levels (**B**) in HepG2 cells after 24 h treatment. Representative immunoblots of the cytosolic content of COX-2 in HepG2 cells (**A**) from three separate experiments are shown. The sequence of the bands corresponds to the sequence of the bars in the graph. β-actin was used as a loading control. The values were calculated as protein levels in comparison to control cells set to 100%. The values (mean ± SEM) for the mRNA levels in HepG2 cells (**B**) were calculated from three separate experiments in comparison to control cells set to 1. * Significantly different from control: DMSO-treated cells, *p* < 0.05. Student’s *t*-test was used to assess the statistical significance.

Since COX-2 is a target gene of NF-κB and is often overexpressed in hepatic cancer cells, its cytosolic protein and mRNA levels were evaluated. Treatment with compounds **5d**, **6c**, and **6d** resulted in reduced COX-2 transcript and protein levels by approximately 25–30% (Figure 5A). Compound **5c** decreased the level of COX-2 transcript without altering its protein level (Figure 5B).

These findings demonstrate that IBU and KET-OAO conjugates in different ways affect NF-κB activity but ultimately reduce its target gene, i.e., the COX-2 expression in HCC cells.

### 2.6. Bead-Based Multiplex Immunoassay Revealed Possible Modulation of Protein Regulating Several Signaling Pathways by Ibuprofen and Ketoprofen—OAO Derivatives Conjugates

The effect of tested compounds on the total, referring to the overall abundance of an examined protein and phosphorylated (active) protein levels of kinases from the MAPKs family, including AKT, c-Jun N-terminal kinase (JNK), p38, extracellular signal-regulated kinase 1/2 (ERK1/2), and ribosomal protein S6 kinase (p70S6K), using a bead-based multiplex immunoassay in liver cancer cells lysates was assessed. These kinases play crucial roles in various cellular processes and signaling pathways, and their dysregulation has been implicated in liver cancer progression.

**Figure 6:**
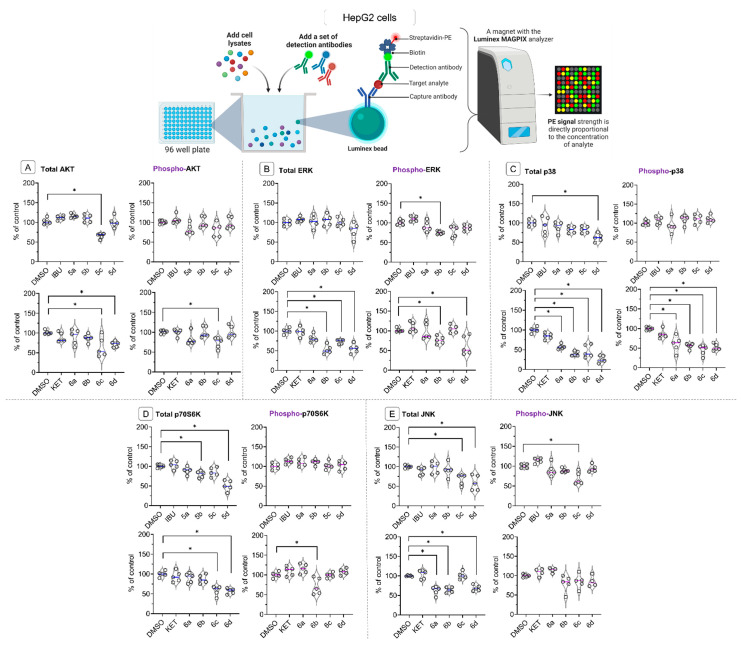
The effect of the IBU, KET, IBU–OAO conjugates (**5a**–**d**) and KET–OAO conjugates (**6a**–**d**) on the total (constitutive) and phosphorylated (active) levels of AKT (**A**), ERK (**B**), p38 (**C**), p70S6K (**D**) and JNK (**E**), measured and calculated on the MAGPIX^®^ system in HepG2 cells, after 24 h incubation with tested compounds at concentrations of 30 µM. The mean values (mean ± SEM) of fluorescence intensity are presented as the fold of control from five independent measurements. * Significantly different from control: DMSO-treated cells, *p* < 0.05. Student’s *t*-test was used to assess the statistical significance.

As shown in Figure 6A, treatment with compounds **5c**, **6c**, and **6d** led to a significant reduction in the total protein levels of AKT and phospho-AKT (only **6c**) in HepG2 cells. The total and phosphorylated protein levels of the extracellular signal-regulated protein kinase (ERK) (Figure 6B) were altered by treatment with KET-OAO derivatives **6b**, **6c**, and **6d**. These changes suggest a potential disruption in ERK-mediated cellular processes, which may also impact liver cancer cell growth and differentiation.

Notably, the levels of p38 (Figure 6C), a stress-activated protein kinase, were strongly diminished by all OAO-KET conjugates, while from OAO-IBU conjugates only morpholide **5d** had an impact on its protein level. Additionally, the total protein level of p70S6K (Figure 6D), a key regulator of protein synthesis and cell growth, was diminished by treatment with IBU-OAO conjugates **5b** and **5d**, as well as OAO-KET derivatives **6c** and **6d**. The reduced level of these proteins might result from their interaction with tested compounds and subsequent destruction and/or diminished expression of genes encoding them. Further studies are required to explain the exact mechanism of the observed changes.

Finally, the protein levels of the c-Jun N-terminal kinase (JNK), which is downstream of AKT and involved in regulating cell proliferation and apoptosis, were also reduced upon treatment with IBU-OAO derivatives **5c** and **5d**, as well as KET-OAO conjugates **5a**, **6b**, and **6d** (Figure 6E). This decrease in JNK protein levels, along with the reduction of p70S6K, indicates a potential impairment of the signaling cascades involved in liver cancer cell growth and survival. Such an assumption is based on the fact that although JNKs, in response to cell stress, activate the pro-apoptotic and autophagy death signals [35], its hyperactivation can promote tumor progression or induce drug-resistance pathways in a wide range of human cancers, including hepatocellular carcinoma [36,37].

Reduction of the p70S6K protein level may lead to cell cycle arrest since this protein plays an essential role in mitogenesis through the progression of the G1 phase of the cell cycle [38,39].

**Figure 7:**
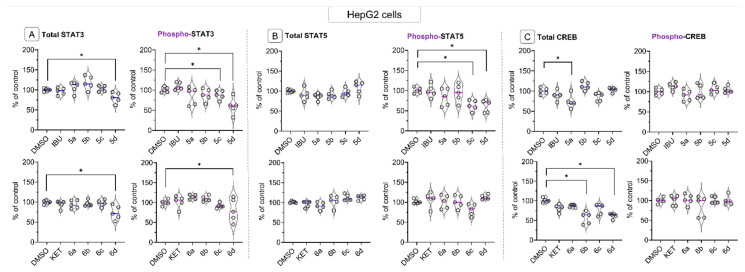
The effect of the IBU, KET, IBU–OAO conjugates (**5a**–**d**) and KET–OAO conjugates (**6a**–**d**) on the total (constitutive) and phosphorylated (active) levels of STAT3 (**A**), STAT5 (**B**) and CREB (**C**) measured and calculated on the MAGPIX^®^ system in HepG2 cells, after 24 h incubation with tested compounds at concentrations of 30 µM. The mean values (mean± SEM) of fluorescence intensity are presented as the fold of control from five independent measurements. * Significantly different from control: DMSO-treated cells, *p* < 0.05. Student’s *t*-test was used to assess the statistical significance.

The effect of IBU-OAO and KET-OAO conjugates on the levels of STAT3, STAT5, and CREB proteins in HepG2 cell lysates was assessed using the same bead-based multiplex immunoassay. The results revealed that compounds **5d** and **6d**, i.e., the OAO morpholide conjugates, led to a reduction in the constitutive/total protein level of STAT3. These compounds, along with compound **5c**, also decreased the level of phosphorylated STAT3 (Figure 7A), indicating a potential inhibition of STAT3 activation. Similarly, the phosphorylated level of STAT5 was reduced by compounds **5c** and **5d**, but there was no significant change in constitutive STAT5 protein level observed (Figure 7B). Both STAT3 and STAT5 transcription factors play a critical role in cell proliferation and survival, and their reduced protein levels indicate a potential disruption of its signaling pathway.

Regarding CREB, the total/constitutive protein level was affected by compound **5a,** and KET-OAO conjugates **6b** and **6d**. However, the phosphorylated form of CREB remained unchanged following treatment with any of the tested compounds (Figure 7C). CREB is another transcription factor involved in cell differentiation and survival, and its altered protein levels suggest a potential modulation of CREB-mediated gene expression in liver cancer cells.

## 3. Discussion

The aim of this study was to investigate the potential of newly synthesized oleanolic acid oxime derivatives (OAO) conjugated with Ibuprofen (IBU-OAO) and Ketoprofen (KET-OAO) as modulators of the signaling pathways, which dysfunction play crucial roles in the liver cancer pathogenesis. Therefore, the study focused on the NF-κB, Nrf2, and MAPKs signaling pathways in immortalized normal hepatocytes and the cells derived from HCC.

Generally, the results confirmed our earlier findings that OAO morpholide is not only the most active among the tested OAO derivatives but also forms the most effective hybrids with NSAIDs [21,22,23,24].

However, it is worth noting the marked differences in the effect on the parameters evaluated between the KET and IBU conjugates and previously tested indomethacin, diclofenac, or aspirin conjugates. While indomethacin and diclofenac conjugate with OAO, particularly substituted with morpholide, significantly increased the toxicity of these NSAIDs [23,24], the conjugation of IBU and KET with OAO derivatives reduced the cytotoxicity of parent compounds in both normal hepatocytes and liver cancer cells.

To a certain extent, a similar effect was observed as a result of the conjugation of aspirin with OAO derivatives. However, in this case, conjugation with aspirin reduced the cytotoxicity of OAO derivatives [21], suggesting the chemopreventive potential of the conjugates. This suggestion was further supported by the activation of the Nrf2-ARE pathway in normal hepatocytes and the reduction of NF-κB activity in both normal and cancer liver cells [22].

Activation of the Nrf2-ARE pathway in normal immortalized hepatocytes was observed in this study as a result of treatment with KET-OAO conjugates demonstrated by increased levels of nuclear Nrf2 protein and enhanced binding to the antioxidant response element (ARE) sequence. Moreover, the increased Nrf2 gene transcript indicated a possible contribution to enhanced Nrf2 activation upregulation of Nrf2 expression and ultimately defense against oxidative or electrophilic stress in these cells.

However, in cancer HepG2 cells, treatment with IBU-OAO or KET-OAO conjugates led rather to a reduction in the level of nuclear Nrf2 protein and binding to ARE sequence, suggesting the tendency to downregulation of this pathway. The reduced mRNA transcript level further supports the suggestion.

As overexpression of Nrf2 due to genetic and epigenetic alterations often occur in cancer cells, including HCC, its reduced expression and activation may prevent enhanced invasiveness potential and chemo- and radioresistance [40]. It has to be stressed that the most potent inducers in THLE-2, i.e., immortalized normal hepatocytes and inhibitors in HepG2 cells, were the hybrids of IBU and KET with OAO substituted with morpholide group.

Therefore, these compounds might be considered chemopreventive agents applicable in HCC prophylaxis and supporting conventional therapy. Interestingly, recently it was found that IBU can induce ferroptosis of glioblastoma cells via downregulation of the Nrf2 signaling pathway [41]. It is possible that a similar effect may occur in HCC cells as a result of treatment with IBU- and KET-OAO derivatives conjugates.

The impact of IBU-OAO and KET-OAO conjugates on the NF-κB pathway, in terms of binding its active subunits p50 and p65 into DNA, and their translocation from the cytosol into the nucleus, was also evaluated. In contrast to the effect of previously tested NSAIDs, particularly diclofenac [23], p50 binding to DNA and the nuclear level were more affected than the p65 NF-κB subunit. The latter was reduced only as a result of treatment with IBU conjugated with OAO morpholide derivative. The p65 subunit is basically responsible for the transcription initiation, while p50 serves as a helper in NF-κB DNA binding [42,43]. Since the disproportionate increases in the active p65 subunits often occur in cancer, this subunit is recognized as an important target for novel drug design [44]. Hence, IBU-OAO morpholide may be worth further studies in this context. However, treatment with KET-OAO conjugates, specifically compounds **6a** (KET-OAO carboxylic derivative) and **6d** (KET-OAO morpholide derivative), decreased the level of phosphorylated NF-κB.

Several studies have revealed the crucial contribution of NF-κB phosphorylation to controlling NF-κB directed transactivation. Moreover, it was shown that NF-κB phosphorylation controls transcription in a gene-specific manner [45]. Therefore, IBU and KET-OAO derivatives conjugates may affect different mechanisms of NF-κB activation. Ultimately it led to reduced expression of its target COX-2 gene, often overexpressed in hepatic cancer cells [46,47]. In this regard, reduced COX-2 transcript and protein levels were observed after treatment with compounds **5d** and **6d,** i.e., conjugates with morpholide OAO derivatives and **6c** (KET-OAO benzyl ester).

Interestingly treatment with compound **6d**, but also **5c** (IBU-OAO benzyl ester) and **6c** led to a significant reduction in the total (constitutive) protein levels of AKT and the case of **6c** also its active phosphorylated form in HepG2. The modulation of the other elements of MAPK signaling pathways, including AKT, ERK, p38, p70S6K, and JNK, was also observed. Notably, the levels of p38, a stress-activated protein kinase, were strongly diminished by all KET-OAO derivatives conjugates and **5d** IBU-OAO morpholide conjugate. Additionally, being a stress-activated kinase, p38 was originally described as a tumor suppressor due to its inhibitory role in RAS-dependent transformation, but later it was shown that it could also act as a tumor promoter. Therefore, its diminished activation indicates its phosphorylated protein level reduction may limit cancer cell proliferation [48].

The c-Jun N-terminal kinase (JNK), which is downstream of AKT and involved in regulating cell proliferation and apoptosis, protein level was also reduced upon treatment with IBU-OAO derivatives, particularly **5d**, as well as KET-OAO conjugates **5a** (IBU-OAO), **6b** (KET-OAO benzyl ester), and **6d** (KET-OAO morpholide).

Diminished AKT activity and related MAPK proteins can have important implications for HCC cells, as AKT signaling is often dysregulated in cancer and contributes to tumor growth and progression. Inhibiting AKT activity, similarly to ERK, p38, p70S6K, and JNK, can potentially lead to reduced cell survival, decreased proliferation, and impaired tumor growth [49,50,51]. Interestingly, several studies have correlated the phosphorylation of ERK and Nrf2 activation [52,53,54]. Moreover, it was shown that the activation of MAPK–Akt and ERK is required for OA-induced activation of Nrf2 in primary rat vascular smooth muscle cells [55].

In this study, such correlation was found in the case of KET-OAO morpholide conjugate, which reduced to the most extent both pathways in HepG2 cells. Finally, the results of the current study revealed that morpholide conjugates, particularly KET hybrid, affect the STAT3, STAT5 activation, and CREB, indicating possible disruption of these signaling pathways associated with cell survival and proliferation [10,56,57].

This suggestion refers particularly to STAT3, which aberrant activation has been reported in nearly 70% of cancers, causing the continuous transcription of cell growth factors and anti-apoptotic molecules, thus playing a crucial role in maintaining cell growth and survival. Diminished activation of STAT3 in concert with MAPKs may significantly limit the growth and survival of HCC cells [58,59].

Overall, the findings of this study demonstrate that IBU-OAO and KET-OAO derivative conjugates, similarly to previously assessed NSAIDs, modulate the key signaling pathways involved in hepatic cancer development. However, although the KET-OAO morpholide seems to be the most potent modulator, their overall effects varied toward specific signaling pathways depending on the OAO derivatives structure. Therefore, selecting the optimal structure for the desired biological is needed.

Importantly, the conjugation of IBU and KET with OAO derivatives reduced their cytotoxicity, which made the conjugates good candidates for the prevention of not only liver cancer but also other diseases with inflammatory origin as safer alternatives to the parent compounds. In cancer therapy, these conjugates might be applied as co-adjuvant reducing unfavorable side effects of conventional drugs. Particularly, by combining these conjugates with existing chemotherapy drugs or treatments, we may enhance the response of chemo- and radiotherapy-resistant HCC cells. Further investigations are warranted to fully exploit the therapeutic potential of these conjugates and to optimize their clinical use in the management of HCC.

## 4. Materials and Methods

### 4.1. Chemistry

General information concerning the performed chemical experiments as well as concerning the elucidation of the synthesized compounds’ chemical structures, is presented in our earlier publication [21]. The purity of the obtained oleanolic acid oxime conjugates with IBU (**5a**–**d**) and KET (**6a**–**d**) was evaluated based on spectra data (NMR) and TLC analysis.

The method involved mixing at room temperature a saturated solution of triterpene oxime (1.0 mmol) in dioxane, with the addition of dicyclohexylcarbodiimide (DCC, 1.5 mmol) and Ibuprofen or Ketoprofen (1.3 mmol). Then the resulting suspension was filtered, and the filtrate was poured into about five times its volume of water. The resulting sticky product of the consistency of a lubricant was extracted with methylene chloride, the organic solution was washed with water, dried, and the solvent was distilled off. After purification of the product on a silica gel column chromatography, an almost colorless product, still of the consistency of a lubricant, was dissolved in ethanol and poured into water but did not form a textured precipitate, but a sticky semi-solid. To obtain the product in the form of a solid, the ethanolic solution of the purified conjugate had to be poured into about 1% brine and left for several days at room temperature. After the product solidified, it was ground, filtered, washed with water, and then dried on air. The conjugates thus obtained were in the form of an amorphous solid with low melting points, except for the compound with Ketoprofen moiety and a morpholine ring (**6d**). This compound had the appearance of an amorphous solid, but at the same time, it was difficult to measure its melting point because the substance melted slowly and in a fairly wide range (although it was chromatographically pure).

**The conjugate of oleanolic acid oxime and Ibuprofen, 3-ibuprofenoxyiminoolean-12-en-28-oic acid (5a):**

**C_43_H_63_NO_4_**. **Mol. mass**: 657.98. **Yield**: 580 mg (88.8%). **M.p.**: 93–96 °C.

**IR** (ν, cm^−1^): 3340 (CH, [CH_3_]_2_CH-CH_2_-C_6_H_4_-CH[CH_3_]-COON=C_-3_), 1730 (C=O, [CH_3_]_2_CH-CH_2_-C_6_H_4_-CH[CH_3_]-COON=C_-3_), 1725 (N=C, [CH_3_]_2_CH-CH_2_-C_6_H_4_-CH[CH_3_]-COON=C_-_**_3_**), 1695 (C=O, -COOH).

**^13^C NMR** (δ, ppm): 183.55 (C_q_, -COOH, C-28), 175.78 (C_q_, [CH_3_]_2_CH-CH_2_-C_6_H_4_-CH-[CH_3_]-COON=C_-3_), 172.43 (C_q_, C-3), 143.24 (C_q_, C-13), 140.42 (C_q_, [CH_3_]_2_CH-CH_2_-C_6_H_4_-CH-[CH_3_]-COON=C_-3_, C-6′), 137.49 (C_q_, [CH_3_]_2_CH-CH_2_-C_6_H_4_-CH-[CH_3_]-COON=C_-3_, C-3′), 137.37 (CH, [CH_3_]_2_CH-CH_2_-C_6_H_4_-CH-[CH_3_]-COON=, C-5′), 129.57 (CH, [CH_3_]_2_CH-CH_2_-C_6_H_4_-CH-[CH_3_]-COON=C_-3_, C-7′), 127.23 (CH, [CH_3_]_2_CH-CH_2_-C_6_H_4_-CH-[CH_3_]-COON=C_-3_, C-4′), 126.83 (CH, [CH_3_]_2_CH-CH_2_-C_6_H_4_-CH-[CH_3_]-COON=C_-3_, C-8′), 122.65 (CH, C-12), 46.72 (C_q_, C-17), 44.90 (CH_2_, [CH_3_]_2_CH-CH_2_-C_6_H_4_-CH-[CH_3_]-COON=C_-3_, C-9′), 41.34 (CH, [CH_3_]_2_CH-CH_2_-C_6_H_4_-CH-[CH_3_]-COON=C_-3_, C-1′), 26.83 (CH, [CH_3_]_2_CH-CH_2_-C_6_H_4_-CH-[CH_3_]-COON=C_-3_, C-10′), 22.29 and 22.24 (CH_3_, [CH_3_]_2_CH-CH_2_-C_6_H_4_-CH-[CH_3_]-COON=C_-3_, C-11′ and C-12′), 14.96 (CH_3_, [CH_3_]_2_CH-CH_2_-C_6_H_4_-CH-[CH_3_]-COON=C_-3_).

**^1^H NMR** (δ, ppm): 7.23 (2H, dd, J = 7.7 and 3.9 Hz, [CH_3_]_2_CH-CH_2_-C_6_H_4_-CH-[CH_3_]-COON=C_-3_, C_4′_-H and C_8′_-H), 7.09 (2H, d, J = 7.0 Hz, [CH_3_]_2_CH-CH_2_-C_6_H_4_-CH-[CH_3_]-COON=C_-3_, C_5′_-H and C_7′_-H), 5.28 (1H, t, J = 3.4 Hz, C_12_-H), 3.86 (1H, quart, J = 7.1 Hz, [CH_3_]_2_CH-CH_2_-C_6_H_4_-CH-[CH_3_]-COON=C_-3_, C_1′_-H), 2.86 (1H, dd, J = 11.7 and 3.2 Hz, C_18_-H_β_), 2.44 (2H, d, J = 7.0 Hz, [CH_3_]_2_CH-CH_2_-C_6_H_4_-CH-[CH_3_]-COON=C_-3_, C_9′_-H_2_), 2.14 (1H, tt, J = 2.5 and 12.1 Hz, [CH_3_]_2_CH-CH_2_-C_6_H_4_-CH-[CH_3_]-COON=C_-3_, C_10′_-H), 1.54 (3H, d, J = 7.1 Hz, [CH_3_]_2_CH-CH_2_-C_6_H_4_-CH-[CH_3_]-COON=C_-3_, C_2′_-H_3_), 1.25 (3H, d, J = 2.4 Hz) and 1.11 (3H, d, J = 3.5 Hz, ([CH_3_]_2_CH-CH_2_-C_6_H_4_-CH-[CH_3_]-COON=C_-3_, C_11′_-H_3_, C_12′_-H_3_), 0.98, 0.96, 0.92, 0.90, 0.89 × 2, 0.87 (5 × 3H + 1 × 6H, 6 × s, 7 × CH_3_ groups).

**The conjugate of methyl oleanolate oxime and Ibuprofen, 3-ibuprofenoxyiminoolean-12-en-28-oic acid methyl ester (5b):**

**C_44_H_65_NO**_4_. **Mol. mass**: 672.00. **Yield**: 620 mg (92.2%). **M.p.**: 68–73 °C.

**IR** (ν, cm^−1^): 3340 (CH, [CH_3_]_2_CH-CH_2_-C_6_H_4_-CH[CH_3_]-COON=C_-3_), 1735 (C=O, [CH_3_]_2_CH-CH_2_-C_6_H_4_-CH[CH_3_]-COON=C_-3_), 1725 (N=C, [CH_3_]_2_CH-CH_2_-C_6_H_4_-CH[CH_3_]-COON=C_-**3**_), 1720 (C=O, -COO-CH_3_).

**^13^C NMR** (δ, ppm): 178.25 (C_q_, -COO-CH_3_, C-28), 175.81 (C_q_, [CH_3_]_2_CH-CH_2_-C_6_H_4_-CH-[CH_3_]-COON=C_-3_), 172.50 (C_q_, C-3), 143.93 (C_q_, C-13), 140.50 (C_q_, [CH_3_]_2_CH-CH_2_-C_6_H_4_-CH-[CH_3_]-COON=C_-3_, C-6′), 137.55 (C_q_, [CH_3_]_2_CH-CH_2_-C_6_H_4_-CH-[CH_3_]-COON=C_-3_, C-3′), 137.48 (CH, [CH_3_]_2_CH-CH_2_-C_6_H_4_-CH-[CH_3_]-COON=, C-5′), 129.27 (CH, [CH_3_]_2_CH-CH_2_-C_6_H_4_-CH-[CH_3_]-COON=C_-3_, C-7′), 127.30 (CH, [CH_3_]_2_CH-CH_2_-C_6_H_4_-CH-[CH_3_]-COON=C_-3_, C-4′), 127.28 (CH, [CH_3_]_2_CH-CH_2_-C_6_H_4_-CH-[CH_3_]-COON=C_-3_, C-8′), 122.04 (CH, C-12), 51.55 (CH_3_, -COO-CH_3_), 46.73 (C_q_, C-17), 45.01 (CH_2_, [CH_3_]_2_CH-CH_2_-C_6_H_4_-CH-[CH_3_]-COON=C_-3_, C-9′), 41.41 (CH, [CH_3_]_2_CH-CH_2_-C_6_H_4_-CH-[CH_3_]-COON=C_-3_, C-1′), 26.95 (CH, [CH_3_]_2_CH-CH_2_-C_6_H_4_-CH-[CH_3_]-COON=C_-3_, C-10′), 22.35 × 2 (CH_3_, [CH_3_]_2_CH-CH_2_-C_6_H_4_-CH-[CH_3_]-COON=C_-3_, C-11′ and C-12′), 15.02 (CH_3_, [CH_3_]_2_CH-CH_2_-C_6_H_4_-CH-[CH_3_]-COON=C_-3_).

**^1^H NMR** (δ, ppm): 7.24 (2H, dd, J = 7.6 and 3.9 Hz, [CH_3_]_2_CH-CH_2_-C_6_H_4_-CH-[CH_3_]-COON=C_-3_, C_4′_-H and C_8′_-H), 7.10 (2H, d, J = 7.1 Hz, [CH_3_]_2_CH-CH_2_-C_6_H_4_-CH-[CH_3_]-COON=C_-3_, C_5′_-H and C_7′_-H), 5.29 (1H, t, J = 3.4 Hz, C_12_-H), 3.85 (1H, quart, J = 7.0 Hz, [CH_3_]_2_CH-CH_2_-C_6_H_4_-CH-[CH_3_]-COON=C_-3_, C_1′_-H), 3.63 (3H, s, -COO-CH_3_), 2.87 (1H, dd, J = 11.8 and 3.2 Hz, C_18_-H_β_), 2.45 (2H, d, J = 7.1 Hz, [CH_3_]_2_CH-CH_2_-C_6_H_4_-CH-[CH_3_]-COON=C_-3_, C_9′_-H_2_), 2.16 (1H, tt, J = 2.5 and 12.0 Hz, [CH_3_]_2_CH-CH_2_-C_6_H_4_-CH-[CH_3_]-COON=C_-3_, C_10′_-H), 1.55 (3H, d, J = 7.0 Hz, [CH_3_]_2_CH-CH_2_-C_6_H_4_-CH-[CH_3_]-COON=C_-3_, C_2′_-H_3_), 1.24 (3H, d, J = 2.4 Hz) and 1.11 (3H, d, 3.5 Hz, ([CH_3_]_2_CH-CH_2_-C_6_H_4_-CH-[CH_3_]-COON=C_-3_, C_11′_-H_3_, C_12′_-H_3_), 1.09, 0.98, 0.94, 0.91, 0.90, 0.89, 0.75 (7 × 3H, 7 × s, 7 × CH_3_ groups).

**The conjugate of benzyl oleanolate oxime and Ibuprofen, 3-ibuprofenoxyiminoolean-12-en-28-oic acid benzyl ester (5c):**

**C_50_H_69_NO_4_**. **Mol. mass**: 748.10. **Yield**: 650 mg (87.0%). **M.p.**: 75–81 °C.

**IR** (ν, cm^−1^): 3340 (CH, [CH_3_]_2_CH-CH_2_-C_6_H_4_-CH[CH_3_]-COON=C_-3_ and -COO-CH_2_-C_6_H_5_), 1735 (C=O, [CH_3_]_2_CH-CH_2_-C_6_H_4_-CH[CH_3_]-COON=C_-3_), 1725 (N=C, [CH_3_]_2_CH-CH_2_-C_6_H_4_-CH[CH_3_]-COON=C_-**3**_), 1705 (C=O, -COO-CH_2_-C_6_H_5_).

**^13^C NMR** (δ, ppm): 177.39 (C_q_, -COO-CH_2_-C_6_H_5_, C-28); 175.86 (C_q_, [CH_3_]_2_CH-CH_2_-C_6_H_4_-CH-[CH_3_]-COON=C_-3_), 172.47 (C_q_, C-3), 143.85 (C_q_, C-13), 140.48 (C_q_, [CH_3_]_2_CH-CH_2_-C_6_H_4_-CH-[CH_3_]-COON=C_-3_, C-6′), 137.59 (C_q_, [CH_3_]_2_CH-CH_2_-C_6_H_4_-CH-[CH_3_]-COON=C_-3_, C-3′), 137.49 (CH, [CH_3_]_2_CH-CH_2_-C_6_H_4_-CH-[CH_3_]-COON=C_-3_, C-5′), 136.42 (C_q_, -COO-CH_2_-C_6_H_5_), 129.28(CH, [CH_3_]_2_CH-CH_2_-C_6_H_4_-CH-[CH_3_]-COON=C_-3_, C-7′), 128.42 (CH × 2, -COO-CH_2_-C_6_H_5_), 128.01 (CH × 2, -COO-CH_2_-C_6_H_5_), 127.93 (CH, -COO-CH_2_-C_6_H_5_), 127.31 (CH, [CH_3_]_2_CH-CH_2_-C_6_H_4_-CH-[CH_3_]-COON=C_-3_, C-4′), 127.30 (CH, [CH_3_]_2_CH-CH_2_-C_6_H_4_-CH-[CH_3_]-COON=C_-3_, C-8′), 122.18 (CH, C-12), 65.95 (CH_2_, -COO-CH_2_-C_6_H_5_), 46.76 (C_q_, C-17), 45.03 (CH_2_, [CH_3_]_2_CH-CH_2_-C_6_H_4_-CH-[CH_3_]-COON=C_-3_, C-9′), 41.45 (CH, [CH_3_]_2_CH-CH_2_-C_6_H_4_-CH-[CH_3_]-COON=C_-3_, C-1′), 26.98 (CH, [CH_3_]_2_CH-CH_2_-C_6_H_4_-CH-[CH_3_]-COON=C_-3_, C-10′), 22.36 (CH_3_ × 2, [CH_3_]_2_CH-CH_2_-C_6_H_4_-CH-[CH_3_]-COON=C_-3_), 15.03 (CH_3_, [CH_3_]_2_CH-CH_2_-C_6_H_4_-CH-[CH_3_]-COON=C_-3_).

**^1^H NMR** (δ, ppm): 7.28 (1H, d, J = 3.6 Hz, -COO-CH_2_-C_6_H_5_), 7.23 (2H, dd, J = 7.6 and 3.9 Hz, [CH_3_]_2_CH-CH_2_-C_6_H_4_-CH-[CH_3_]-COON=C_-3_, C_4′_-H and C_8′_-H), 7.16 (4H, dd, J = 8.2 and 7.1 Hz, -COO-CH_2_-C_6_H_5_), 7.12–7.06 (2H, m, [CH_3_]_2_CH-CH_2_-C_6_H_4_-CH-[CH_3_]-COON=C_-3_, C_5′_-H and C_7′_-H), 5.33 (1H, t, J = 3.5 Hz, C_12_-H), 5.11 (2H, d, J = 12.6 Hz, -COO-CH_2_-C_6_H_5_), 3.85 (1H, quart, J = 7.0 Hz, [CH_3_]_2_CH-CH_2_-C_6_H_4_-CH-[CH_3_]-COON=C_-3_, C_1′_-H), 2.95 (1H, dd, J = 4.0 and 13.7 Hz, C_18_-H_β_), 2.46 (2H, d, J = 7.0 Hz, [CH_3_]_2_CH-CH_2_-C_6_H_4_-CH-[CH_3_]-COON=C_-3_, C_9′_-H_2_), 2.14 (1H, tt, J = 2.5 and 12.0 Hz, [CH_3_]_2_CH-CH_2_-C_6_H_4_-CH-[CH_3_]-COON=C_-3_, C_10′_-H), 1.56 (3H, d, J = 7.0 Hz, [CH_3_]_2_CH-CH_2_-C_6_H_4_-CH-[CH_3_]-COON=C_-3_, C_2′_-H_3_), 1.24 (3H, d, J = 2.4 Hz) and 1.12 (3H, d, 3.5 Hz, ([CH_3_]_2_CH-CH_2_-C_6_H_4_-CH-[CH_3_]-COON=C_-3_, C_11′_-H_3_, C_12′_-H_3_), 0.97, 0.95, 0.92 × 3, 0.90, 0.64 (4 × 3H + 1 × 9H, 5 × s, 7 × CH_3_ groups).

**The conjugate of morpholide of oleanolic acid oxime and Ibuprofen, 3-ibuprofenoxyiminoolean-12-en-28-oic acid morpholide (5d):**

**C_47_H_70_N_2_O_4_**. **Mol. mass**: 727.08. **Yield**: 662 mg (91.1%). **M.p.**: 100–105 °C.

**IR** (ν, cm^−1^): 3335 (CH, [CH_3_]_2_CH-CH_2_-C_6_H_4_-CH[CH_3_]-COON=C_-3_), 1730 (C=O, [CH_3_]_2_CH-CH_2_-C_6_H_4_ CH[CH_3_]-COON=C_-3_), 1720 (N=C, [CH_3_]_2_CH-CH_2_-C_6_H_4_-CH[CH_3_]-COON=C**_-3_**), 1630 (C=O, -C(O)Mor); 995 (C–N, -C(O)Mor); Mor = morpholine ring.

**^13^C NMR** (δ, ppm): 175.79 (C_q_, [CH_3_]_2_CH-CH_2_-C_6_H_4_-CH-[CH_3_]-COON=C_-3_), 175.07 (C_q_, -C(O)Mor); 172.41 (C_q_, C-3), 144.78 (C_q_, C-13), 140.43 (C_q_, [CH_3_]_2_CH-CH_2_-C_6_H_4_-CH-[CH_3_]-COON=C_-3_, C-6′), 137.49 (C_q_, [CH_3_]_2_CH-CH_2_-C_6_H_4_-CH-[CH_3_]-COON=C_-3_, C-3′), 137.39 (CH, [CH_3_]_2_CH-CH_2_-C_6_H_4_-CH-[CH_3_]-COON=C_-3_, C-5′), 129.58 (CH, [CH_3_]_2_CH-CH_2_-C_6_H_4_-CH-[CH_3_]-COON=C_-3_, C-7′), 127.25 (CH, [CH_3_]_2_CH-CH_2_-C_6_H_4_-CH-[CH_3_]-COON=C_-3_, C-4′), 127.20 (CH, [CH_3_]_2_CH-CH_2_-C_6_H_4_-CH-[CH_3_]-COON=C_-3_, C-8′), 121.20 (CH, C-12), 66.89 × 2 (CH_2_ × 2, Mor), 47.35 (C_q_, C-17), 46.05 and 41.60 (CH_2_ × 2, Mor), 44.91 (CH_2_, [CH_3_]_2_CH-CH_2_-C_6_H_4_-CH-[CH_3_]-COON=C_-3_, C-9′), 41.86 (CH, [CH_3_]_2_CH-CH_2_-C_6_H_4_-CH-[CH_3_]-COON=C_-3_, C-1′), 26.90 (CH, [CH_3_]_2_CH-CH_2_-C_6_H_4_-CH-[CH_3_]-COON=C_-3_, C-10′), 22.29 and 22.22 (CH_3_ × 2, [CH_3_]_2_CH-CH_2_-C_6_H_4_-CH-[CH_3_]-COON=C_-3_), 15.01 (CH_3_, [CH_3_]_2_CH-CH_2_-C_6_H_4_-CH-[CH_3_]-COON=C_-3_); Mor = morpholine ring.

**^1^H NMR** (δ, ppm): 7.24 (2H, dd, J = 7.7 and 3.9 Hz, [CH_3_]_2_CH-CH_2_-C_6_H_4_-CH-[CH_3_]-COON=C_-3_, C_4′_-H and C_8′_-H), 7.11 (2H, d, J = 7.1 Hz, [CH_3_]_2_CH-CH_2_-C_6_H_4_-CH-[CH_3_]-COON=C_-3_, C_5′_-H and C_7′_-H), 5.28 (1H, t, J = 3.5 Hz, C_12_-H), 3.85 (1H, quart, J = 7.1 Hz, [CH_3_]_2_CH-CH_2_-C_6_H_4_-CH-[CH_3_]-COON=C_-3_, C_1′_-H), 3.70–3.58 (8H, m, Morph), 3.10 (2H, d, J = 11.4 Hz, C_18_-H_β_), 2.45 (2H, d, J = 7.2 Hz, [CH_3_]_2_CH-CH_2_-C_6_H_4_-CH-[CH_3_]-COON=C_-3_, C_9′_-H_2_), 2.15 (1H, tt, J = 2.5 and 12.0 Hz, [CH_3_]_2_CH-CH_2_-C_6_H_4_-CH-[CH_3_]-COON=C_-3_, C_10′_-H), 1.56 (3H, d, J = 7.0 Hz, [CH_3_]_2_CH-CH_2_-C_6_H_4_-CH-[CH_3_]-COON=C_-3_, C_2′_-H_3_), 1.24 (3H, d, J = 2.4 Hz) and 1.10 (3H, d, J = 3.5 Hz, ([CH_3_]_2_CH-CH_2_-C_6_H_4_-CH-[CH_3_]-COON=C_-3_, C_11′_-H_3_, C_12′_-H_3_), 1.33, 1.21, 1.19, 1.07, 1.00, 0.99, 0.92 (7 × 3H, 7 × s, 7 × CH_3_ groups); Mor = morpholine ring.

**The conjugate of oleanolic acid oxime and Ketoprofen, 3-ketoprofenoxyiminoolean-12-en-28-oic acid (6a):**

**C_46_H_59_NO_5_**. **Mol. mass**: 705.98. **Yield**: 659 mg (93.3%). **M.p.**: 80–84 °C.

**IR** (ν, cm^−1^): 3335 (CH, C_6_H_5_-CO-C_6_H_4_-CH[CH_3_]-COON=C-_3_), 1735 (C=O, C_6_H_5_-CO-C_6_H_4_-CH[CH_3_]-COON=C-_3_), 1725 (N=C, C_6_H_5_-CO-C_6_H_4_-CH[CH_3_]-COON=C-_3_), 1695 (C=O, -COOH).

**^13^C NMR** (δ, ppm): 196.50 (C_q_, C_6_H_5_-CO-C_6_H_4_-CH[CH_3_]-COON=C_-3_, C-9′), 183.55 (C_q_, -COOH, C-28), 175.94 (C_q_, C_6_H_5_-CO-C_6_H_4_-CH[CH_3_]-COON=C_-3_), 171.84 (C_q_, C-3), 143.24 (C_q_, C-13), 140.66 (C_q_, C_6_H_5_-CO-C_6_H_4_-CH[CH_3_]-COON=C_-3_, C-3′), 137.91 (C_q_, C_6_H_5_-CO-C_6_H_4_-CH[CH_3_]-COON=C_-3_, C-5′), 137.48 (C_q_, C_6_H_5_-CO-C_6_H_4_-CH[CH_3_]-COON=C_-3_, C-10′), 132.50 (CH, C_6_H_5_-CO-C_6_H_4_-CH[CH_3_]-COON=C_-3_, C-13′), 132.04 (CH, C_6_H_5_-CO-C_6_H_4_-CH[CH_3_]-COON=C_-3_, C-8′), 130.05 (CH × 2, C_6_H_5_-CO-C_6_H_4_-CH[CH_3_]-COON=C_-3_, C-11′ and C-15′), 129.36 (CH, C_6_H_5_-CO-C_6_H_4_-CH[CH_3_]-COON=C_-3_, C-7′), 129.13 (CH, C_6_H_5_-CO-C_6_H_4_-CH[CH_3_]-COON=C_-3_, C-4′), 128.65 (CH, C_6_H_5_-CO-C_6_H_4_-CH[CH_3_]-COON=C_-3_, C-6′), 128.31 (CH × 2, C_6_H_5_-CO-C_6_H_4_-CH[CH_3_]-COON=C_-3_, C-12 and C-14′), 122.64 (CH, C-12), 46.72 (C_q_, C-17), 44.53 (CH, C_6_H_5_-CO-C_6_H_4_-CH[CH_3_]-COON=C_-3_, C-1′), 18.55 (CH_3_, C_6_H_5_-CO-C_6_H_4_-CH[CH_3_]-COON=C_-3_, C-2′)

**^1^H NMR** (δ, ppm): 7.80 (2H, dd, J = 3.5 and 1.8 Hz, C_6_H_5_-CO-C_6_H_4_-CH-[CH_3_]-COON=C_3_, C_4′_-H and C_11′_-H), 7.69 (1H, d, J = 7.7 Hz, C_6_H_5_-CO-C_6_H_4_-CH-[CH_3_]-COON=C_3_, C_6′_-H), 7.62 (2H, t, J = 6.1 Hz, C_6_H_5_-CO-C_6_H_4_-CH-[CH_3_]-COON=C_3_, C_12′_-H and C_14′_-H), 7.55–7.43 (3H, m, C_6_H_5_-CO-C_6_H_4_-CH-[CH_3_]-COON=C_3_, C_13′_-H, C_7′_-H and C_8′_-H), 5.28 (1H, t, J = 3.4 Hz, C_12_-H), 3.99 (1H, quart., J = 7.0 Hz, C_6_H_5_-CO-C_6_H_4_-CH-[CH_3_]-COON=C_3_), 2.85 (1H, dd, J = 11.7 and 3.2 Hz, C_18_-H_β_), 1.61 (3H, d, J = 7.1 Hz, C_6_H_5_-CO-C_6_H_4_-CH-[CH_3_]-COON=C_3_), 1.11, 1.00, 0.99, 0.95 × 2, 0.92, 0.76 (5 × 3H + 1 × 6H, 6 × s, 7 × CH_3_ group).

**Conjugate of methyl oleanolate oxime and Ketoprofen, 3-ketoprofenoxyiminoolean-12-en-28-oic acid methyl ester (6b):**

**C_47_H_61_NO_5_**. **Mol. mass**: 720.01. **Yield**: 667 mg (92.7%). **M.p.**: 72–75 °C.

**IR** (ν, cm^−1^): 3330 (CH, C_6_H_5_-CO-C_6_H_4_-CH[CH_3_]-COON=C-_3_), 1730 (C=O, C_6_H_5_-CO-C_6_H_4_-CH[CH_3_]-COON=C-_3_), 1725 (N=C, C_6_H_5_-CO-C_6_H_4_-CH[CH_3_]-COON=C**-_3_**), 1720 (C=O, -COO-CH_3_).

**^13^C NMR** (δ, ppm): 196.48 (C_q_, C_6_H_5_-CO-C_6_H_4_-CH[CH_3_]-COON=C_-3_, C-9′), 178.26 (C_q_, -COO-CH_3_, C-28), 175.94 (C_q_, C_6_H_5_-CO-C_6_H_4_-CH[CH_3_]-COON=C_-3_), 171.85 (C_q_, C-3), 143.94 (C_q_, C-13), 140.69 (C_q_, C_6_H_5_-CO-C_6_H_4_-CH[CH_3_]-COON=C_-3_, C-3′), 137.91 (C_q_, C_6_H_5_-CO-C_6_H_4_-CH[CH_3_]-COON=C_-3_, C-5′), 137.50 (C_q_, C_6_H_5_-CO-C_6_H_4_-CH[CH_3_]-COON=C_-3_, C-10′), 132.53 (CH, C_6_H_5_-CO-C_6_H_4_-CH[CH_3_]-COON=C_-3_, C-13′), 132.04 (CH, C_6_H_5_-CO-C_6_H_4_-CH[CH_3_]-COON=C_-3_, C-8′), 130.07 (CH × 2, C_6_H_5_-CO-C_6_H_4_-CH[CH_3_]-COON=C_-3_, C-11′ and C-15′), 129.38 (CH, C_6_H_5_-CO-C_6_H_4_-CH[CH_3_]-COON=C_-3_, C-7′), 129.03 (CH, C_6_H_5_-CO-C_6_H_4_-CH[CH_3_]-COON=C_-3_, C-4′), 128.55 (CH, C_6_H_5_-CO-C_6_H_4_-CH[CH_3_]-COON=C_-3_, C-6′), 128.34 (CH × 2, C_6_H_5_-CO-C_6_H_4_-CH[CH_3_]-COON=C_-3_, C-12 and C-14′), 122.04 (CH, C-12), 51.56 (CH_3_, COO-CH_3_), 46.10 (C_q_, C-17), 44.50 (CH, C_6_H_5_-CO-C_6_H_4_-CH[CH_3_]-COON=C_-3_, C-1′), 18.54 (CH_3_, C_6_H_5_-CO-C_6_H_4_-CH[CH_3_]-COON=C_-3_, C-2′).

**^1^H NMR** (δ, ppm): 7.81 (2H, dd, J = 3.5 and 1.9 Hz, C_6_H_5_-CO-C_6_H_4_-CH-[CH_3_]-COON=C_3_, C_4′_-H and C_11′_-H), 7.69 (1H, d, J = 7.6 Hz, C_6_H_5_-CO-C_6_H_4_-CH-[CH_3_]-COON=C_3_, C_6′_-H), 7.61 (2H, t, J = 6.0 Hz, C_6_H_5_-CO-C_6_H_4_-CH-[CH_3_]-COON=C_3_, C_12′_-H and C_14′_-H), 7.55–7.42 (3H, m, C_6_H_5_-CO-C_6_H_4_-CH-[CH_3_]-COON=C_3_, C_13′_-H, C_7′_-H and C_8′_-H), 5.30 (1H, t, J = 3.4 Hz, C_12_-H), 3.98 (1H, quart., J = 7.0 Hz, C_6_H_5_-CO-C_6_H_4_-CH-[CH_3_]-COON=C_3_), 3.64 (3H, s, -COO-CH_3_), 2.88 (1H, dd, J = 13.6 and 3.7 Hz, C_18_-H_β_), 1.62 (3H, d, J = 7.2 Hz, C_6_H_5_-CO-C_6_H_4_-CH-[CH_3_]-COON=C_3_), 1.11, 1.00, 0.99, 0.95 × 2, 0.92, 0.76 (5 × 3H + 1 × 6H, 6 × s, 7 × CH_3_ group).

**The conjugate of benzyl oleanolate oxime and Ketoprofen, 3-ketoprofenoxyiminoolean-12-en-28-oic acid benzyl ester (6c):**

**C_43_H_65_NO_5_**. **Mol. mass**: 796.10. **Yield**: 672 mg (84.4%). **M.p.**: 59–63 °C.

**IR** (ν, cm^−1^): 3335 (CH, C_6_H_5_-CO-C_6_H_4_-CH[CH_3_]-COON=C-_3_), 2900 (CH, -COO-CH_2_-C_6_H_5_), 1740 (C=O, C_6_H_5_-CO-C_6_H_4_-CH[CH_3_]-COON=C-_3_), 1725 (N=C, C_6_H_5_-CO-C_6_H_4_-CH[CH_3_]-COON=C**-_3_**), 1705 (C=O, -COO-CH_2_-C_6_H_5_).

**^13^C NMR** (δ, ppm): 196.88 (C_q_, C_6_H_5_-CO-C_6_H_4_-CH[CH_3_]-COON=C_-3_, C-9′), 177.41 (C_q_, -COO-CH_2_-C_6_H_5_, C-28); 176.10 (C_q_, C_6_H_5_-CO-C_6_H_4_-CH[CH_3_]-COON=C_-3_), 172.02 (C_q_, C-3), 143.90 (C_q_, C-13), 140.60 (C_q_, C_6_H_5_-CO-C_6_H_4_-CH[CH_3_]-COON=C_-3_, C-3′), 137.57 (C_q_, C_6_H_5_-CO-C_6_H_4_-CH[CH_3_]-COON=C_-3_, C-5′), 137.47 (C_q_, C_6_H_5_-CO-C_6_H_4_-CH[CH_3_]-COON=C_-3_, C-10′), 136.40(C_q_, -COO-CH_2_-C_6_H_5_), 132.64 (CH, C_6_H_5_-CO-C_6_H_4_-CH[CH_3_]-COON=C_-3_, C-13′), 132.08 (CH, C_6_H_5_-CO-C_6_H_4_-CH[CH_3_]-COON=C_-3_, C-8′), 130.17 (CH × 2, C_6_H_5_-CO-C_6_H_4_-CH[CH_3_]-COON=C_-3_, C-11′ and C-15′), 129.37 (CH, C_6_H_5_-CO-C_6_H_4_-CH[CH_3_]-COON=C_-3_, C-7′), 129.13 (CH, C_6_H_5_-CO-C_6_H_4_-CH[CH_3_]-COON=C_-3_, C-4′), 128.65 (CH, C_6_H_5_-CO-C_6_H_4_-CH[CH_3_]-COON=C_-3_, C-6′), 128.38 (CH × 2, -COO-CH_2_-C_6_H_5_), 128.09 (CH × 2, -COO-CH_2_-C_6_H_5_), 127.84 (CH, -COO-CH_2_-C_6_H_5_), 128.29 (CH × 2, C_6_H_5_-CO-C_6_H_4_-CH[CH_3_]-COON=C_-3_, C-12 and C-14′), 122.16 (CH, C-12), 65.89 (CH_2_, -COO-CH_2_-C_6_H_5_), 46.67 (C_q_, C-17), 44.53 (CH, C_6_H_5_-CO-C_6_H_4_-CH[CH_3_]-COON=C_-3_, C-1′), 18.59 (CH_3_, C_6_H_5_-CO-C_6_H_4_-CH[CH_3_]-COON=C_-3_, C-2′).

**^1^H NMR** (δ, ppm): 7.80 (2H, dd, J = 3.5 and 1.9 Hz, C_6_H_5_-CO-C_6_H_4_-CH-[CH_3_]-COON=C_3_, C_4′_-H and C_11′_-H), 7.67 (1H, d, J = 7.6 Hz, C_6_H_5_-CO-C_6_H_4_-CH-[CH_3_]-COON=C_3_, C_6′_-H), 7.59 (2H, t, J = 6.0 Hz, C_6_H_5_-CO-C_6_H_4_-CH-[CH_3_]-COON=C_3_, C_12′_-H and C_14′_-H), 7.54–7.44 (3H, m, C_6_H_5_-CO-C_6_H_4_-CH-[CH_3_]-COON=C_3_, C_13′_-H, C_7′_-H and C_8′_-H), 7.30 (1H, d, J = 3.6 Hz, -COO-CH_2_-C_6_H_5_), 7.15 (4H, dd, J = 8.1 and 7.1 Hz, -COO-CH_2_-C_6_H_5_), 7.12–7.06 (2H, m, -COO-CH_2_-C_6_H_5_), 5.33 (1H, t, J = 3.4 Hz, C_12_-H), 5.10 (2H, d, J = 12.6 Hz, -COO-CH_2_-C_6_H_5_), 3.98 (1H, quart., J = 7.0 Hz, C_6_H_5_-CO-C_6_H_4_-CH-[CH_3_]-COON=C_3_), 2.93 (1H, dd, J = 4.0 and 13.7 Hz, C_18_-H_β_), 1.62 (3H, d, J = 7.2 Hz, C_6_H_5_-CO-C_6_H_4_-CH-[CH_3_]-COON=C_3_), 1.13, 1.11, 1.04, 0.99, 0.92, 0.91, 0.90 (7 × 3H, 7 × s, 7 CH_3_ groups).

**The conjugate of morpholide of oleanolic acid oxime and Ketoprofen, 3-ketoprofenoxyiminoolean-12-en-28-oic acid morpholide (6d):**

**C_50_H_66_N_2_O_5_**. **Mol. mass**: 775.08. **Yield**: 684 mg (88.2%). **M.p**.: ---.

**IR** (ν, cm^−1^): 3330 (CH, C_6_H_5_-CO-C_6_H_4_-CH[CH_3_]-COON=C-_3_), 1735 (C=O, C_6_H_5_-CO-C_6_H_4_-CH[CH_3_]-COON=C-_3_), 1720 (N=C, C_6_H_5_-CO-C_6_H_4_-CH[CH_3_]-COON=C**-_3_**), 1625 (C=O, -C(O)Mor); 995 (C–N, -C(O)Mor); Mor = morpholine ring.

**^13^C NMR** (δ, ppm): 196.36 (C_q_, C_6_H_5_-CO-C_6_H_4_-CH[CH_3_]-COON=C_-3_, C-9′), 175.93 (C_q_, C_6_H_5_-CO-C_6_H_4_-CH[CH_3_]-COON=C_-3_), 175.10 (C_q_, -C(O)Mor); 171.81 (C_q_, C-3), 144.72 (C_q_, C-13), 140.48 (C_q_, C_6_H_5_-CO-C_6_H_4_-CH[CH_3_]-COON=C_-3_, C-3′), 137.92 (C_q_, C_6_H_5_-CO-C_6_H_4_-CH[CH_3_]-COON=C_-3_, C-5′), 137.29 (C_q_, C_6_H_5_-CO-C_6_H_4_-CH[CH_3_]-COON=C_-3_, C-10′), 132.53 (CH, C_6_H_5_-CO-C_6_H_4_-CH[CH_3_]-COON=C_-3_, C-13′), 131.27 (CH, C_6_H_5_-CO-C_6_H_4_-CH[CH_3_]-COON=C_-3_, C-8′), 129.98 (CH × 2, C_6_H_5_-CO-C_6_H_4_-CH[CH_3_]-COON=C_-3_, C-11′ and C-15′), 129.32 (CH, C_6_H_5_-CO-C_6_H_4_-CH[CH_3_]-COON=C_-3_, C-7′), 129.01 (CH, C_6_H_5_-CO-C_6_H_4_-CH[CH_3_]-COON=C_-3_, C-4′), 128.70 (CH, C_6_H_5_-CO-C_6_H_4_-CH[CH_3_]-COON=C_-3_, C-6′), 128.27 (CH × 2, C_6_H_5_-CO-C_6_H_4_-CH[CH_3_]-COON=C_-3_, C-12 and C-14′), 121.18 (CH, C-12), 66.99, 66.87 (CH_2 ×_ 2, Mor), 47.33 (C_q_, C-17), 46.22, 41.84 (CH_2_ × 2, Mor), 44.38 (CH, C_6_H_5_-CO-C_6_H_4_-CH[CH_3_]-COON=C_-3_, C-1′), 18.48 (CH_3_, C_6_H_5_-CO-C_6_H_4_-CH[CH_3_]-COON=C_-3_, C-2′); Mor = morpholine ring.

**^1^H NMR** (δ, ppm): 7.79 (2H, dd, J = 3.5 and 1.9 Hz, C_6_H_5_-CO-C_6_H_4_-CH-[CH_3_]-COON=C_3_, C_4′_-H, and C_11′_-H), 7.67 (1H, d, J = 7.7 Hz, C_6_H_5_-CO-C_6_H_4_-CH-[CH_3_]-COON=C_3_, C_6′_-H), 7.59 (2H, t, J = 6.1 Hz, C_6_H_5_-CO-C_6_H_4_-CH-[CH_3_]-COON=C_3_, C_12′_-H and C_14′_-H), 7.54–7.46 (3H, m, C_6_H_5_-CO-C_6_H_4_-CH-[CH_3_]-COON=C_3_, C_13′_-H, C_7′_-H, and C_8′_-H), 5.28 (1H, t, J = 3.5 Hz, C_12_-H), 4.04 (1H, quart., J = 7.0 Hz, C_6_H_5_-CO-C_6_H_4_-CH-[CH_3_]-COON=C_3_), 3.70–3.58 (8H, m, Morph), 3.08 (2H, d, J = 11.4 Hz, C_18_-H_β_), 1.60 (3H, d, J = 7.2 Hz, C_6_H_5_-CO-C_6_H_4_-CH-[CH_3_]-COON=C_3_), 1.08, 1.04, 0.98, 0.97, 0.93, 0.90, 0.75 (7 × 3H, 7 × s, 7 × CH_3_ groups); Mor = morpholine ring.

### 4.2. Biological Assays

#### 4.2.1. Cell Culture and Viability Assay

HepG2 (ATCC HB 8065) and THLE-2 (ATCC CRL-2706) cells were provided by the American Type Culture Collection (ATCC, Manassas, VA, USA). HepG2 cells were maintained in Dulbecco’s Modified Eagle’s Medium (DMEM, Sigma-Aldrich, Saint Louis, MO, USA) containing 10% fetal bovine serum (FBS, EURx, Gdansk, Poland) and 1% antibiotics solution (Sigma-Aldrich, Saint Louis, MO, USA), while THLE-2 were cultured in BEGM, supplemented with Bullet Kit (Lonza, Cologne, Germany) and 10% FBS, 5 ng/mL EGF, 70 ng/mL phosphoethanolamine at 37 °C, in a humidified, 5% CO2 atmosphere. To assess the effect of IBU, KET, and their OAO conjugates on measured parameters, 1 × 10^6^ cells were seeded per 100 mm culture dish. After 24 h of initial incubation, the cells were treated with 30 µM concentrations of IBU, KET, and their OAO conjugates and 0.1% dimethyl sulfoxide (DMSO) control solution. Incubation lasted for 24 h, and the cells were harvested.

The effect of tested compounds on cell viability was assessed by the MTT assay, following the standard protocol. Briefly, THLE-2 and HepG2 cells were seeded (10^4^ per well) in 96-well plates. After 24 h of preincubation in a complete medium, compounds were added in various concentrations, and cells were incubated for 24 h. Later, cells were washed twice with phosphate-buffered saline (PBS) and further incubated for 4 h with a medium containing 0.5 mg/mL 3-(4,5-dimethylthiazol-2-yl)-2,5-diphenyl-2H-tetrazolium bromide (MTT). Then, the formazan crystals were dissolved in acidic isopropanol, and the absorbance was measured at 570 nm and 690 nm. All experiments were repeated three times. In all following experiments, we used non-toxic concentrations of compounds (with viability level above 70%): 30 μM of IBU, KET, and their OAO conjugates (**5a**–**d** and **6a**–**d**).

#### 4.2.2. Nuclear, Cytosolic, and Total Protein Lysates Preparation

Nuclear and cytosolic fractions were prepared according to the manufacturer’s protocol, and the subcellular extracts from HepG2 and THLE-2 cells were prepared using the Nuclear/Cytosol Fractionation Kit (BioVision Research, Milpitas, CA, USA). Lysates were prepared using Radioimmunoprecipitation assay (RIPA) buffer with the addition of protease inhibitors (Sigma-Aldrich, USA). Protein concentration was assessed, and the samples were stored at −80 °C for future downstream applications.

#### 4.2.3. Total RNA Isolation, cDNA Synthesis, and Quantitative Real-Time PCR (R-T PCR)

The extraction of total RNA was performed by the GeneMatrix Universal DNA/RNA/Protein Purification Kit (EURx, Gdańsk, Poland). Subsequently, samples were subjected to reverse transcription by the RevertAid First Strand cDNA Synthesis Kit (Thermo Fisher Scientific, Waltham, MA, USA), according to the manufacturer’s instructions. 

For the quantitative R-T PCR analyses, the Maxima SYBR Green Kit (Fermentas Inc., Waltham, MA, USA) and the BioRad Chromo4 thermal cycler (BioRad Laboratories, Hercules, CA, USA) were used. Protocol started with 5 min enzyme activation at 95 °C, followed by 40 cycles of 95 °C for 15 s, 56 °C for 20 s, and 72 °C for 40 s, and the final elongation at 72 °C for 5 min. Melting curve analysis was used for amplicon verification. The expression of TBP (TATA box binding protein) and PBGD (porphobilinogen deaminase) was used to normalize data. The Pfaffl comparative method was used for fold-change quantification. Primers were designed using the Beacon Designer software 7.9 and subjected to a BLAST search to minimize unspecific binding. Only the primer pairs that generated intron-spanning amplicons were selected. The primers’ sequences used to analyze Nrf2, NF-кBp65, NF-кBp50, COX-2, TBP, and PBGD genes are listed in Table 1.

#### 4.2.4. Western Blot Analysis

Cytosolic extracts for COX-2 and β-actin, or nuclear extracts for Nrf2, NF-кB p65, NF-кB p50, and lamin protein detection, were separated on 12% or 10% SDS-PAGE slab gels. β-actin and lamin were used as loading control. Proteins were transferred to the nitrocellulose Immobilon P membrane. After blocking for 2 h with 10% skimmed milk, proteins were probed with primary antibodies against Nrf2, NF-кB p65, NF-кB p50, COX-2, β-actin, and lamin. Alkaline phosphatase AP-labeled anti-rabbit IgG, anti-goat IgG, and anti-mouse IgG secondary antibodies (BioRad Laboratories, Hercules, CA, USA) were used in the staining reaction. Bands were visualized by AP Conjugate Substrate Kit NBT/BCIP (BioRad Laboratories, Hercules, CA, USA). The amount of immunoreactive products in each lane was determined using ChemiDoc Imaging System (BioRad Laboratories, Hercules, CA, USA). Values were calculated as relative absorbance units (RQ) per mg of protein and expressed as a percentage of control.

#### 4.2.5. Nrf2 and NF-ĸB Binding Assay

Nrf2, NF-κB p50, and NF-κB p65 activation were assessed by the enzymatic immunoassay (Transcription Factor ELISA Assay Kit Active Motif, Waterloo, Belgium) according to the manufacturer’s instructions. Activated Nrf2 was evaluated based on the amount of Nrf2 contained in the DNA-binding complex to the ARE sequence. The oligonucleotides containing the ARE consensus-binding site (5′-GTCACAGTGACTCAGCAGAATCTG-3′) for Nrf2 were immobilized on microplates as bait. Whereas activated NF-ĸB was measured as the amount of p65 and p50 subunits held in the DNA-binding complex. The oligonucleotides containing (5′-GGGACTTTCC-3′), a consensus site for NF-κB, were immobilized on microplates as bait. Nuclear fractions were incubated with oligonucleotides for 1 h, wells were washed, and DNA-bound subunits were detected by the specific primary antibody and a secondary antibody conjugated with the HRP. The results were expressed as the normalized level of absorbance (OD450 nm per mg of protein).

#### 4.2.6. Bead-Based Immunoassay on the Luminex MAGPIX Instrument

Magnetic bead-based immunoassay was performed on the Luminex-MAGPIX multiplex immunoassay system. Data were analyzed with Milliplex Analyst 5.1 software (EMD Millipore, Burlington, MA, USA). The panel we performed included quantitation of the following constitutive (total) and phosphorylated (active) proteins in the lysates of HepG2 cells, AKT, ERK, p38, p70S6K, JNK, STAT3, STAT5, and CREB according to the manufacturer’s instructions. The magnetic bead panel with high-sensitivity antibodies was obtained from Merck, Germany. A multiplex test based on microspheres using Luminex^®^ xMAP^®^ technology with different fluorescent colors was detected on a compatible MAGPIX^®^ camera. Lysates were suspended in MILLIPLEX^®^ MAP buffer. The bead suspension was added to each well of a 96-well plate; samples were added into the wells and incubated overnight at 2–8 °C on a shaker protected from light. Control samples for HepG2 cells were lysates run as blank wells (containing all assay components minus protein); these were also added to the same plate and mixed with 25 μL of 1 × bead mix. The plate was washed with 2X buffer, and then 1X MILLIPLEX^®^ MAP Detection Antibodies were added. After shaking for 1 h at room temperature, the antibodies were removed, and 1X MILLIPLEX^®^ MAP Streptavidin/Phycoerythrin (SAPE) was added. Then the MILLIPLEX^®^ MAP Amplification Buffer was added to each well and shaken for 15 min. The beads were suspended in MILLIPLEX^®^ MAP buffer, and each microsphere was identified with a MAGPIX^®^ Luminex Analyzer, and the results were calculated from the reporters’ fluorescent signals. Mean fluorescence intensities (MFI) were used to measure the relative total protein and relative phosphorylation levels of different targets and quantified with the xPonent 4.2 software (Luminex Corporation, Austin, TX, USA). The raw MFI results for the tested protein levels were converted relative to the control of DMSO-treated cells.

### 4.3. Statistical Analysis

Statistical analysis and graphs were calculated and prepared using GraphPad Prism (GraphPad Software 9, San Diego, CA, USA), assuming the significance level of changes as *p* < 0.05. Student’s *t*-test was used to assess the statistical significance between the experimental and control groups.

## 5. Conclusions

Our study highlighted the distinct effects of OAO conjugates with Ibuprofen and Ketoprofen on liver cancer-related signaling pathways, NF-κB, Nrf2, and MAPK. The IBU-OAO and KET-OAO morpholide conjugates (**5d** and **6d**) emerged as the most active and effective hybrids. These conjugates present potential targets for novel drug design and may contribute to reducing invasiveness and chemoresistance in liver cancer cells. Further studies, including in vivo studies, are warranted to explore their application in liver cancer treatment.

## Figures and Tables

**Table 1:** Primers used in R-T PCR.

Gene	Forward Primer	Reverse Primer
*Nrf2*	5′ATTGCTACTAATCAGGCTCAG	5′GTTTGGCTTCTGGACTTGG
*NF-ĸB p50*	5′ATCATCCACCTTCATTCTCAA	5′AATCCTCCACCACATCTTCC
*NF-ĸB p65*	5′CGCCTGTCCTTTCTCATC	5′ACCTCAATGTCCTCTTTCTG
*COX-2*	5′CCTGTGCCTGATGATTGC	5′CAGCCCGTTGGTGAAAGC
*PBGD*	5′TCAGATAGCATACAAGAGACC	5′TGGAATGTTACGAGCAGTG
*TBP*	5′GGCACCACTCCACTGTATC	5′GGGATTATATTCGGCGTTTCG

## Data Availability

The data are contained within this article.
